# Limonoid and Steroidal Saponin from *Azadirachta indica*

**DOI:** 10.1007/s13659-014-0042-2

**Published:** 2014-11-08

**Authors:** Lu Liu, Yun-Li Zhao, Gui-Guang Cheng, Ying-Ying Chen, Xu-Jie Qin, Chang-Wei Song, Xing-Wei Yang, Ya-Ping Liu, Xiao-Dong Luo

**Affiliations:** 1 State Key Laboratory of Phytochemistry and Plant Resources in West China, Kunming Institute of Botany, Chinese Academy of Sciences, Kunming, 650201 People’s Republic of China; 2 University of Chinese Academy of Sciences, Beijing, 100049 People’s Republic of China; 3 Yunnan Institute of Food Safety, Kunming University of Science and Technology, Kunming, 650500 People’s Republic of China

**Keywords:** *Azadirachta indica*, Limonoid, Steroidal saponin, Antibacterial activities

## Abstract

A new limonoid, 17-(5-methoxy-2-oxofuran-3-yl)-28-deoxonimbolide (**1**), and a new C21 steroidal saponin, 2*α*,4*α*-dihydroxy-pregn-5-en-16-one-3*α*-*O*-d-glucopyranoside (**2**), together with 11 known compounds were isolated from the methanol extract of the leaves of *Azadirachta indica*. The structures were elucidated by means of spectroscopic analysis and putative biosynthetic origins. All the compounds were evaluated for their antibacterial activities against six bacterial strains.

**Electronic supplementary material:**

The online version of this article (doi:10.1007/s13659-014-0042-2) contains supplementary material, which is available to authorized users.

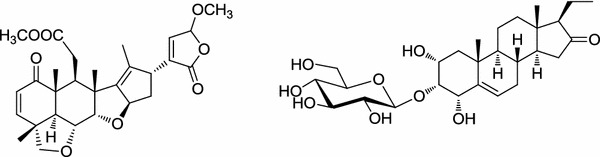

## Introduction

Herbal medicines are widely used and formed as an integral part of primary health care in many countries [1–3], and some are used to treat fungal diseases, which may constitute a reservoir of antifungal substances. In recent years, a number of antifungal agents are currently used in antifungal therapies with clinical practice, such as griseofulvin and terbinafine [4, 5]. These antifungals are isolated from fungi or are synthetized, and searching for new antifungal substances from plants is still challenging [6].

**Fig. 1:**
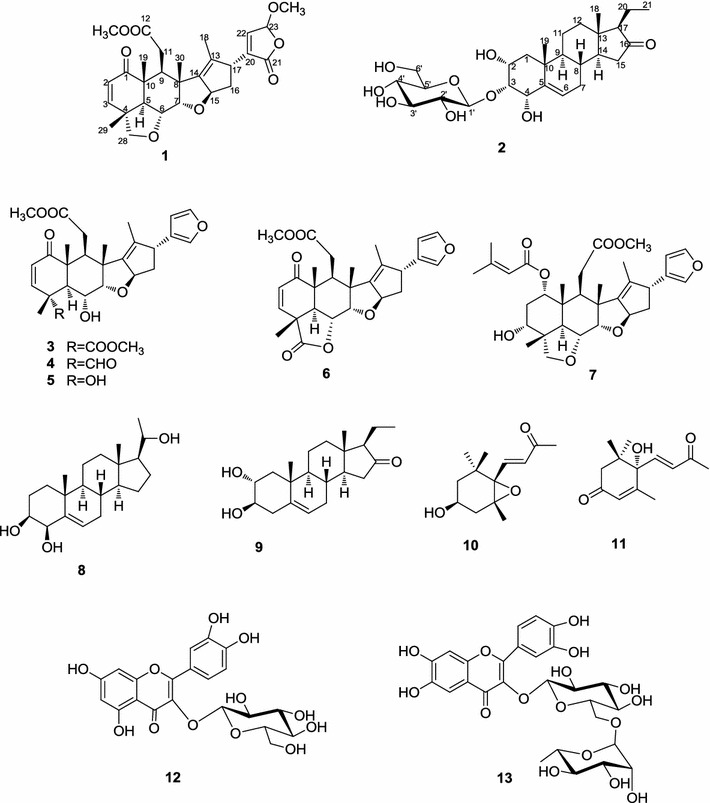
Chemical structures of compounds **1**–**13**

*Azadirachta indica* (neem tree), indigenous to India, belongs to the Meliaceae family, are widely cultivated throughout the tropics and subtropics [7]. In view of therapeutic and bioactive importance of the plant, it has attracted the attention of investigators all over the world to research bioactive compounds [8–10]. Antibacterial effects of neem extract have been demonstrated against some Gram-negative and Gram-positive microorganisms, including *Streptococcus mutans*, *S. faecalis*, *Escherichia coli* and *Pseudomonas florescence* pathogenic strains [11, 12]. In a continuation of our studies on its constituents [8–10], a new limonoid, 17-(5-methoxy-2-oxofuran-3-yl)-28-deoxonimbolide (**1**) and a new C21 steroid saponin, 2*α*,4*α*-dihydroxy-pregn-5-en-16-one-3*α*-*O*-d-glucopyranose (**2**), together with 6-deacetylnimbin (**3**) [13], 6-deacetylnimbinal (**4**) [13], nimbandiol (**5**) [14], nimbolide (**6**) [13], 2′,3′-dehydrosalannol (**7**) [15], 3*β*,4*β*,20*α*-trihydroxy-5-pregnen (**8**) [16], 2*α*,3*β*-dihydroxy-5-pregnen-16-one (**9**) [17], (+)-dehydro-vomifoliol (**10**) [18], 3*β*-hydroxy-5*α*,6*α*-epoxy-7-megastigmen-9-one (**11**) [19], quercetin-3-glucopyranside (**12**) [20] and quercetin-3-glu (6→1) rha (**13**) [21] were isolated from the methanol extract of the leaves of *A. indica* (Fig. 1). The structures were elucidated by means of spectroscopic analysis. All the compounds were evaluated for their antibacterial activities against six bacterial strains.

## Results and discussion

Compound **1** exhibited a molecular formula of C_28_H_34_O_8_ by HR-EI-MS (*m/z* 498.2246 [M]^+^), indicating 12 degrees of unsaturation. The UV maximum at 240 nm indicated the existence of *α*,*β*-unsaturated ketone system, while the IR spectrum showed the absorption bands of *γ*-lactone (1759 cm^−1^), ester C=O (1728 cm^−1^), and conjugated cyclohexenone (1631 cm^−1^) chromophoric groups. Its ^1^H NMR spectrum showed three olefinic proton signals at *δ*_H_ 7.04 (d, *J* = 9.7 Hz), 5.86 (d, *J* = 9.7 Hz) and 6.82 (s), four methyl signals at *δ*_H_ 1.72 (s), 1.14 (s), 1.33 (s) and 1.29 (s), two methoxyl signals at *δ*_H_ 3.63 (s) and 3.50 (s).

The ^13^C NMR and DEPT spectra of **1** displayed 28 carbons signals comprising two ester carbonyl groups, one ketone carbonyl group, three double bonds, three quaternary carbons, seven methines (four oxygenated ones), three methylenes (one oxygenated carbon), six methyls (Table 1). These aforementioned data suggested that compound **1** is a nimbolide-type limonoid, structurally similar to 28-deoxonimbolide [13]. The *α,β*-unsaturated-*γ*-lactone moiety was suggested on the basis of four carbons at *δ*_C_ 139.4 (s, C-20), 171.3 (s, C-21), 142.3 (d, C-22), and 102.3 (d, C-23), and the corresponding protons at *δ*_H_ 6.82 (s, H-22) and 5.73 (s, H-23). The assumption was supported by the ^1^H-^1^H COSY spin system of H-22/H-23, and by the HMBC correlations of *δ*_H_ 6.82 (s, H-22) and 5.73 (s, H-23) with *δ*_C_ 171.3 (s, C-21) and 139.4 (s, C-20). In addition, the HMBC correlation of methoxyl protons at *δ*_H_ 3.50 with a hemiketal group at *δ*_C_ 102.3 (d, C-23), suggested the methoxyl group was placed at C-23. In the ROESY spectrum, correlations of Me-29 with H-6, of Me-30 with H-7 positioned *β*-orientation for both H-6 and H-7. The ROESY correlations of H-5/H-9/H-15, indicated the *α*-orientation of H-15. Other parts of **1** were identical to those of 28-deoxonimbolide, as further confirmed by detailed analysis of 2D NMR spectra of **1**. Thus, the structure of **1** was elucidated as shown (Fig. 1), and named as 17-(5-methoxy-2-oxofuran-3-yl)-28-deoxonimbolide.

**Table 1:** ^1^H and ^13^C NMR data for compounds **1** and **2** (*δ* in ppm, *J* in Hz)

No.	1^a^		No.	2^b^	
	*δ*_H_	*δ*_C_		*δ*_H_	*δ*_C_
1		202.4 (s)	1	1.09 (m), 2.14 (m)	46.5 (t)
2	5.86 (d, 9.7)	130.1 (d)	2	4.11 (m)	66.2 (d)
3	7.04 (d, 9.7)	152.1 (d)	3	3.44 (dd, 3.5, 3.6)	85.5 (d)
4		46.1 (s)	4	4.37 (d, 3.5)	76.6 (d)
5	2.74 (d, 12.6)	48.9 (d)	5		142.4 (s)
6	4.10 (dd, 12.6, 3.4)	72.3 (d)	6	5.76 (t, 3.7)	129.4 (d)
7	4.21 (d, 3.4)	85.4 (d)	7	2.12 (m)	33.1 (t)
8		50.6 (s)	8	1.75 (m)	31.6 (d)
9	2.53 (t, 5.4)	41.2 (d)	9	1.18 (m)	51.8 (d)
10		46.1 (s)	10		38.5 (s)
11	2.35 (dd, 5.4, 16.4)	32.5 (t)	11	1.65 (m)	21.2 (t)
	3.25 (dd, 5.4, 16.4)		12	1.47 (m), 1.96 (m)	38.8 (t)
12		173.5 (s)	13		42.9 (s)
13		132.4 (s)	14	1.54 (m)	51.8 (d)
14		148.7 (s)	15	1.81 (m), 2.25 (m)	39.3 (t)
15	5.32 (m)	87.1 (d)	16		221.8 (s)
16	2.10 (m), 2.18 (m)	39.9 (t)	17	1.77 (m)	66.0 (d)
17	3.54 (m)	48.5 (d)	18	0.74 (s)	13.6 (q)
18	1.72 (s)	13.1 (q)	19	1.29 (s)	22.3 (q)
19	1.14 (s)	14.5 (q)	20	1.30 (m), 1.60 (m)	18.6 (t)
20		139.4 (s)	21	1.03 (t, 7.5)	13.8 (q)
21		171.3 (s)	1′	4.41 (d, 7.7)	102.5 (d)
22	6.82 (s)	142.3 (d)	2′	3.29 (m)	74.9 (d)
23	5.73 (s)	102.3 (d)	3′	3.38 (m)	77.6 (d)
28	3.69 (d, 7.3),	79.3 (t)	4′	3.29 (m)	71.5 (d)
	3.79 (d, 7.3)		5′	3.29 (m)	78.1 (d)
29	1.33 (s)	20.5 (q)	6′	3.64 (m), 3.84 (m)	62.6 (t)
30	1.29 (s)	17.1 (q)			
11-COOCH_3_	3.63 (s)	51.7 (q)			
23-OCH_3_	3.50 (s)	56.2 (q)			

^a^Measured in CDCl_3_^1^H and ^13^C NMR were recorded at 400 and 100 MHz, respectively

^b^Measured in CD_3_OD ^1^H and ^13^C NMR were recorded at 600 and 150 MHz, respectively

**Fig. 2:**
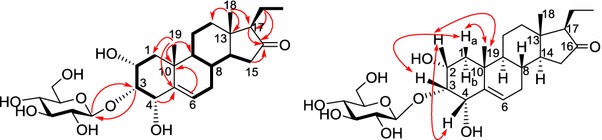
Selected COSY (), HMBC () and ROESY () correlations of compound **2**

Compound **2** was assigned a molecular formula of C_27_H_42_O_9_, by its HR-EI-MS peak at *m/z* 510.2833 ([M]^+^ calcd. for 510.2829), in combination with ^1^H, ^13^C NMR and DEPT data, corresponding to 7 degrees of unsaturation. IR absorptions bands at 3428 and 1631 cm^−1^ revealed the existence of OH and C=C groups. Signals of two high-field quaternary carbons (*δ*_C_ 38.5, 42.9) in the ^13^C NMR spectrum, along with two singlet methyls (*δ*_H_ 0.74, 1.29) and a triplet methyl (*δ*_H_ 1.03) (Table 1) in the ^1^H NMR spectrum, suggested that compound **2** was a pregnane derivative [15]. Correlations of *δ*_H_ 1.77 (H-17) with *δ*_C_ 221.8 (s, C-16), and of *δ*_H_ 1.54 (H-14) with *δ*_C_ 221.8 (s, C-16) in the HMBC spectrum assigned C-16 as a ketone (Fig. 2). The HMBC correlations of *δ*_H_ 4.37 (d, *J* = 3.5 Hz, H-4)/*δ*_C_ 142.4 (s, C-5), of *δ*_H_ 1.29 (s, Me-19)/*δ*_C_ 142.4 (s, C-5), and of *δ*_H_ 5.76 (t, *J* = 3.7 Hz, H-6)/*δ*_C_ 38.5 (s, C-10) suggested the existence of double bond between at C-5 and C-6. The ^1^H-, ^13^C-NMR, and DEPT spectra displayed a d-glucopyranosyl unit on the basis of an anomeric proton signal at *δ*_H_ 4.41 (d, *J* = 7.7 Hz, H-1′), an methylene signals at *δ*_H_ 3.64 (m, H-6′) and 3.84 d (m, H-6′), four additional protons between *δ*_H_ 3.29 and *δ*_H_ 3.38; as well as an anomeric C-atom (*δ*_C_ 102.5, d), an methylene group (*δ*_C_ 66.9, t), and other four CH groups [*δ*_C_ 74.9 (d), 77.6 (d), 71.5 (d), 78.1 (d)]. The sugar unit was further verified as d-glucopyranose by GC analysis of its corresponding trimethylsilylated l-cysteine adduct after acidic hydrolysis of **2** [22]. Correlations of *δ*_H_ 3.44 (dd, *J* = 3.5, 3.6 Hz, H-3)/*δ*_C_ 102.5 (d, C-1′), and of *δ*_H_ 4.41 (d, *J* = 7.7 Hz, H-1′)/*δ*_C_ 85.5 (d, C-3) in the HMBC spectrum indicated the sugar unit was linked at C-3. In the ROESY spectrum, correlations of *δ*_H_ 3.44 (Me-19)/4.11 (H-2)/4.37 (H-4), and of *δ*_H_ 3.44 (Me-19)/1.09 (H-1a)/3.44 (H-3) positioned *β*-orientation for H-2, H-3 and H-4, and *α*-orientation for OH-2, O-Glc-3 and OH-4 (Fig. 2). Thus, the structure of **2** was elucidated as shown, and named as 2*α*,4*α*-dihydroxy-pregn-5-en-16-one-3*α*-*O*-d-glu-copyranoside.

All the compounds were evaluated for their antibacterial activities against *Escherichia coli* ATCC 11775, *Enterococcus faecalis* ATCC 10541, *Klebsiella pneumonia* ATCC 13883, *Pseudomonas aeruginosa* ATCC 27853, *Staphylococcus aureus* ATCC 25922, and *Salmonella enterica* ATCC 13076. Norfloxacin was used as the positive control. The results showed that compounds **1** and **2** exhibited strong antibacterial activities against some bacterial strains, equivalent to norfloxacin with MIC values of 0.78 μg/mL (Table 2). And the other results (MIC values) are summarized in Table 2.

**Table 2:** Minimum inhibitory concentration (MIC, μg/mL) of compounds **1**–**13** against six bacterial strains

	*Escherichia coli* ATCC 11775	*Enterococcus faecalis* ATCC 10541	*Klebsiella pneumonia* ATCC 13883	*Pseudomonas aeruginosa* ATCC 27853	*Staphylococcus aureus* ATCC 25922	*Salmonella enterica* ATCC 13076
**1**	0.78	6.25	0.78	100	0.78	NA
**2**	0.78	6.25	100	25	NA	6.25
**3**	1.56	NA	6.25	6.25	1.56	NA
**4**	1.56	100	1.56	0.78	25	NA
**5**	6.25	100	1.25	25	0.78	100
**6**	1.56	25	1.56	6.25	1.56	NA
**7**	6.25	25	0.78	1.56	1.56	NA
**8**	25	NA	6.25	0.78	1.56	6.25
**9**	NA	100	6.25	1.56	6.25	NA
**10**	NA	NA	25	6.25	25	6.25
**11**	1.56	NA	1.56	1.56	100	6.25
**12**	0.78	0.78	25	6.25	25	25
**13**	0.78	NA	1.56	25	6.25	NA
Norfloxacin	0.19	0.78	0.78	0.19	0.78	0.78

*NA* not active (MIC >100 μg/mL)

## Experimental Section

### General Experimental Procedures

Optical rotations were obtained with a Jasco P-1020 Automatic Digital Polariscope. UV spectrum was measured with a Shi madzu UV2401PC spectrometer. IR spectra were obtained on a Bruker FT-IR Tensor-27 infrared spectrophotometer with KBr pellets. ^1^H, ^13^C, and 2D NMR spectra were recorded on a Bruker AM-400, Bruker DRX-500 NMR and Bruker DRX-600 spectrometer with TMS as internal standard. ESI-MS and HR-EI-MS analysis were carried out on Waters Xevo TQS and Waters AutoSpec Premier P776 mass spectrometers, respectively. Semi-preparative HPLC was performed on an Agilent 1100 HPLC with a ZORBAX SB-C18 (9.4 × 250 mm) column. Silica gel (100–200 and 200–300 mesh, Qingdao Marine Chemical Co. Ltd., P.R. China), Sephadex LH-20 (GE Healthcare Bio-Xciences AB), and MCI gel (75–150 μm, Mitsubishi Chemical Corporation, Tokyo, Japan) were used for column chromatography. Fractions were monitored by TLC (GF 254, Qingdao Marine Chemical Co. Ltd., P.R. China), and spots were visualized by 10 % H_2_SO_4_-ethanol reagent.

### Plant Material

The leaves of *A. indica* were collected from Xishuangbanna county, Yunnan province, China, in June 2012, and were identified by Dr. Jian Liu. A voucher specimen (Luo20120620) has been deposited at the State Key Laboratory of Phytochemistry and Plant Resources in West China, Kunming Institute of Botany, Chinese Academy of Sciences.

### Extraction and Isolation

The leaves of *A. indica* A. Juss. (10 kg) were powdered and extracted with MeOH (30 L percolation, 12 h × 3) to yield a MeOH extract (1745 g). The MeOH extract was suspended in H_2_O (4 L) and partitioned successively with EtOAc (3 L × 3) to yield an EtOAc fraction (320 g) and a H_2_O-soluble fraction (1425 g). The EtOAc fraction was subjected to silica gel column chromatography eluted with a gradient solvent system of chloroform/acetone (from 1:0 to 0:1, v/v) to afford five fractions (A–E) on the basis of TLC detection. First of all, fraction D (200 g, MeOH/H_2_O) was chromatographed on MCI column for removing the color, and then by RP-18 column chromatography (MeOH–H_2_O) to yield subfractions a–c. Each subfraction was purified by RP C-18 (MeOH–H_2_O) silica gel column (chloroform/acetone system), preparative thick layer chromatography (PTLC), and Sephadex LH-20 column (MeOH) successively, to yield compounds **1** (15 mg), **3** (950 mg), **4** (500 mg), **5** (4.1 g), **6** (560 mg), and **7** (6.8 g). Fraction E (93 g) was subjected to MCI column, silica gel column chromatography, PTLC, and Sephadex LH-20 column in turns to give compounds **2** (8 mg), **8** (4.1 g), **9** (15 mg), **10** (20 mg), **11** (13 mg), **12** (50 mg) and **13** (70 mg).

### 17-(5-Methoxy-2-oxofuran-3-yl)-28-deoxonimbolide (**1**)

White powder; [α]_D_^21^ +112.3 (*c* 0.11, CHCl_3_); UV (CHCl_3_) *λ*_max_ (log ε) 240.8 (3.63); IR (KBr) *ν*_max_ 3428, 2933, 2877, 2854, 1759, 1728, 1631, 1384 cm^−1^; ^1^H and ^13^C NMR data see Table 1; positive ESIMS *m/z* 521 [M+Na]^+^; HREIMS *m/z* 498.2246 (calcd for C_28_H_34_O_8_ [M]^+^, 498.2254).

### 2*α*,4*α*-Dihydroxy-pregn-5-en-16-one-3*α*-*O*-d-glucopyranoside (2)

White powder; [α]_D_^21^ −112.1 (*c* 0.10, MeOH); IR (KBr) *ν*_max_ 3428, 2933, 2877, 2854, 1738, 1631, 1384 cm^−1^; ^1^H and ^13^C NMR data see Table 1; positive ESIMS *m/z* 533 [M+Na]^+^; HREIMS *m/z* 510.2833 (calcd for C_27_H_42_O_9_ [M]^+^, 510.2829).

### Acidic Hydrolysis of **2** and GC analysis

Compound **2** (2 mg) was hydrolyzed with 2 M HCl/dioxane (1:1, 10 mL) under reflux for 3 h. The reaction mixture was partitioned between CHCl_3_ and H_2_O. The aqueous layer was neutralized with 2 M NaOH and then dried to give a sugar. The sugar was dissolved in anhydrous pyridineand (1 mL) and reacted with l-cysteine methyl ester hydrochloride (1.5 mg) stirred at 60 °C for 1.5 h. Then trimethylsilylimidazole (1.0 mL) was added to the reaction mixture, and it was kept at 60 °C for 30 min. The mixture (4 μL) was subjected to GC analysis, run on an HP 5890 gas chromatograph (Agilent) with a quartz capillary column (30 mm × 0.32 mm × 0.25 μm): H_2_ flame ionization detector, column temp 180–280 °C at 3 °C/min, carrier gas N_2_ (1 mL/min), injector and detector temp 250 °C, split ratio 1:50. Peak of the hydrolysate was detected by comparison with retention time of authentic samples of d-glucose after treatment with trimethyl-chlorsilan (TMCS) in pyridine. The absolute configurations of the sugar residue was determined to be d-glucose (*t*_R_ 19.01 min).

### Antimicrobial Assays

The antibacterial assay of compounds **1**–**13** was evaluated against *E. coli* ATCC 11775, *E. faecalis* ATCC 10541, *K. pneumonia* ATCC 13883, *P. aeruginosa* ATCC 27853, *S. aureus* ATCC 25922, and *S. enterica* ATCC 13076. All the bacterial strains were obtained from the American Type Culture Collection (Rockville, USA). The antibacterial assay was carried out as described in the literature [23]. The preparation of bacterial inocula was done by using 18 hold overnight bacterial cultures prepared in Nutrient Agar. A few colonies of bacteria were collected aseptically with a sterile loop and introduced into 10 mL of sterile 0.90 % saline solution. The concentration of the suspension was then standardized by adjusting the optical density to 0.10 at 600 nm, corresponding to bacterial cell suspension of 108 colony- forming units/mL (CFU/mL) [24]. This cell suspension was diluted 100 times to obtain 106 CFU/mL before use. The compounds were dissolved in DMSO and then added to bacteria suspension to obtain the final concentration of 5 % (v/v) DMSO or less. Serial twofold dilutions from 200 μg/mL of the compounds were performed in 96-well micro-titer plates. Each well contained 100 μL of sample of each concentration. Then each well was infunded 100 μL of the bacterial suspension. The final concentration range of the test compounds was 100–0.781 μg/mL, and the plates were incubated at 37 °C for 24 h. After incubation, the wells were examined for growth of microorganisms by measuring optical density (OD) value of the wells. Each experiment was repeated three times and Norfloxacin, bacteria suspension of 5 % (v/v) DMSO were used as a positive control and a blank control, respectively. By comparing to OD values, we can point out MIC values of these compounds among the selected concentration range.

## Electronic supplementary material

Below is the link to the electronic supplementary material.

(DOC 16118 kb)
