# Supplementary material for: Limonoid and Steroidal Saponin from Azadirachta indica
Source: Nat Prod Bioprospect. 2014 Nov 8;4(6):335–40. doi: 10.1007/s13659-014-0042-2 (PMC4250567; doi:10.1007/s13659-014-0042-2)

**Supporting Information**

**Limonoid and Steroidal saponin from *Azadirachta indica***

Lu Liu ^a, b^, Yun-Li Zhao ^a^, Gui-Guang Cheng ^c^, Ying-Ying Chen ^a, b^, Xu-Jie Qin ^a, b^, Chang-Wei Song ^a, b^, Xing-Wei Yang ^a, b^, Ya-Ping Liu ^a^, *, and Xiao-Dong Luo ^a^,*

^a^ State Key Laboratory of Phytochemistry and Plant Resources in West China, Kunming Institute of Botany, Chinese Academy of Sciences, Kunming 650201, China

^b^ University of Chinese Academy of Sciences, Beijing 100049, China

^c^ Yunnan Institute of Food Safety, Kunming University of Science and Technology, Kunming, 650500, P. R. China

* To whom correspondence should be addressed.

E-mail: [xdluo@mail.kib.ac.cn](mailto:xdluo@mail.kib.ac.cn); [liuyaping@mail.kib.ac.cn](mailto:liuyaping@mail.kib.ac.cn)

**Figure 1S.** ^1^H NMR spectrum of compound **1**.

**Figure 2S.** ^13^C NMR spectrum of compound **1**.

**Figure 3S.** HSQC spectrum of compound **1**.

**Figure 4S.** ^1^H-^1^H COSY spectrum of compound **1**.

**Figure 5S.** HMBC spectrum of compound **1**.

**Figure 6S.** ROESY spectrum of compound **1**.

**Figure 7S.** HREIMS spectrum of compound **1**.

**Figure 8S.** ^1^H NMR spectrum of compound **2**.

**Figure 9S.** ^13^C NMR spectrum of compound **2**.

**Figure 10S.** HSQC spectrum of compound **2**.

**Figure 11S.** ^1^H-^1^H COSY spectrum of compound **2**.

**Figure 12S.** HMBC spectrum of compound **2**.

**Figure 13S.** ROESY spectrum of compound **2**.

**Figure 14S.** HREIMS spectrum of compound **2**.

**Figure 1S.** ^1^H NMR spectrum of compound **1**.


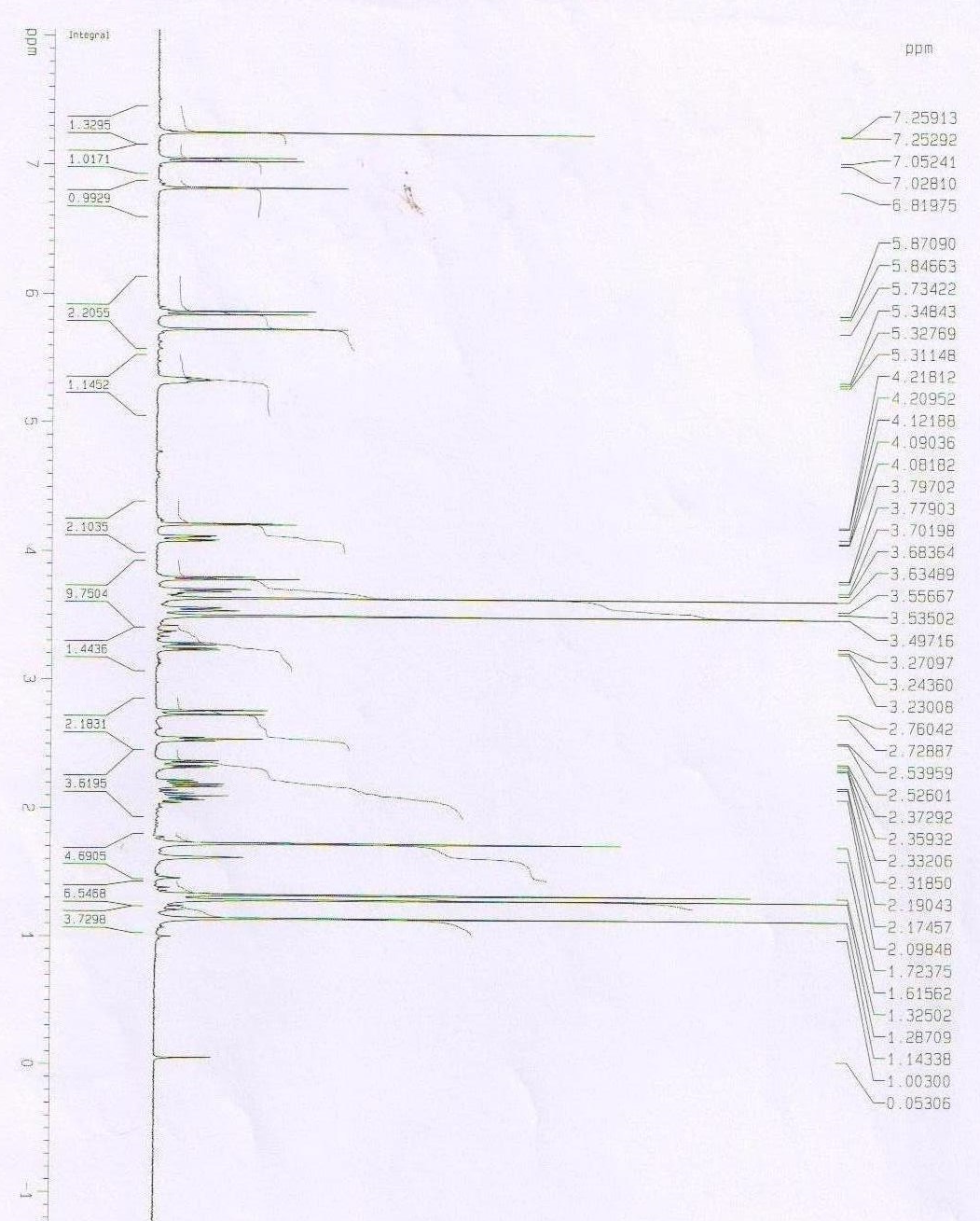


**Figure 2S.** ^13^C NMR spectrum of compound **1**.


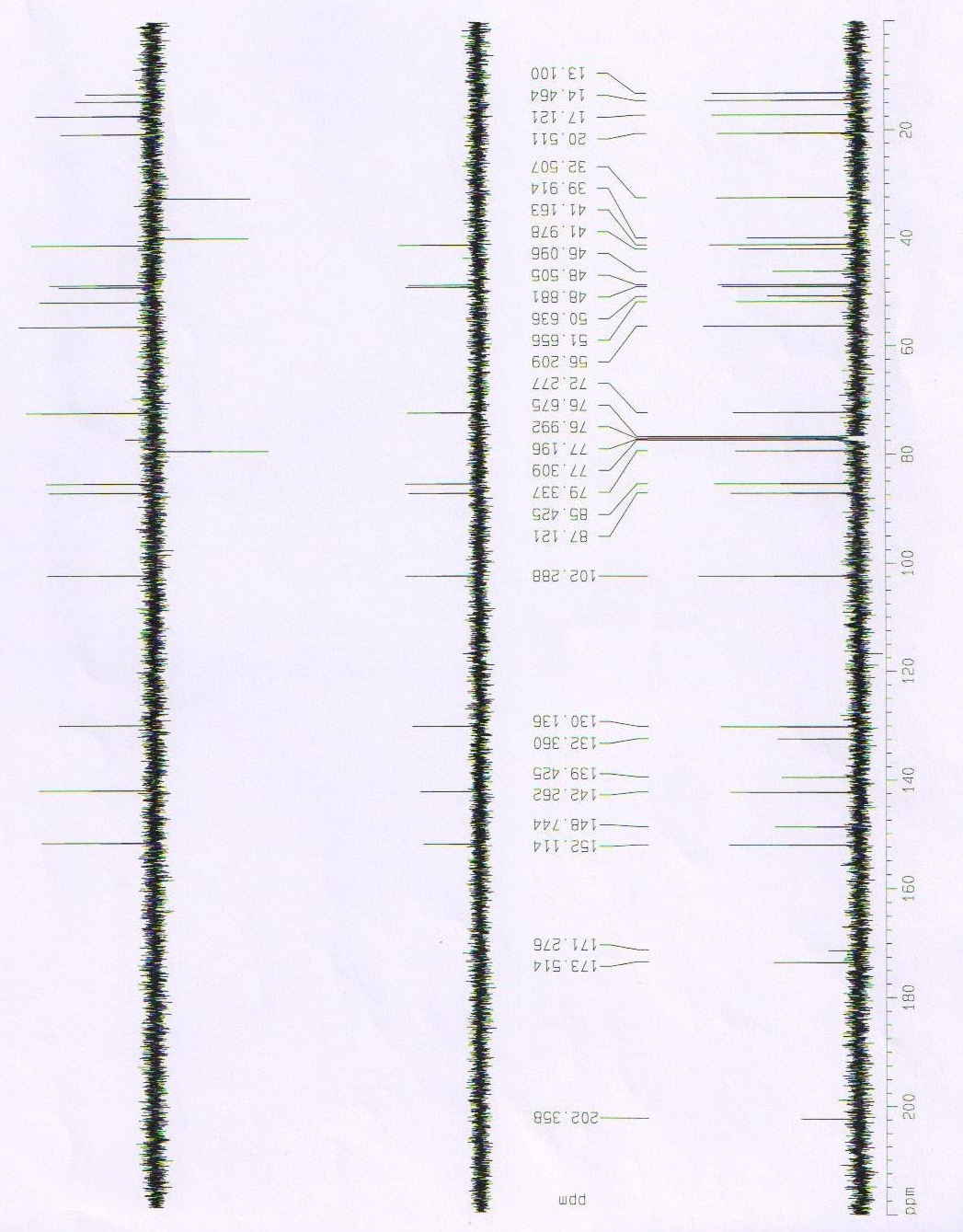


**Figure 3S.** HSQC spectrum of compound **1**.


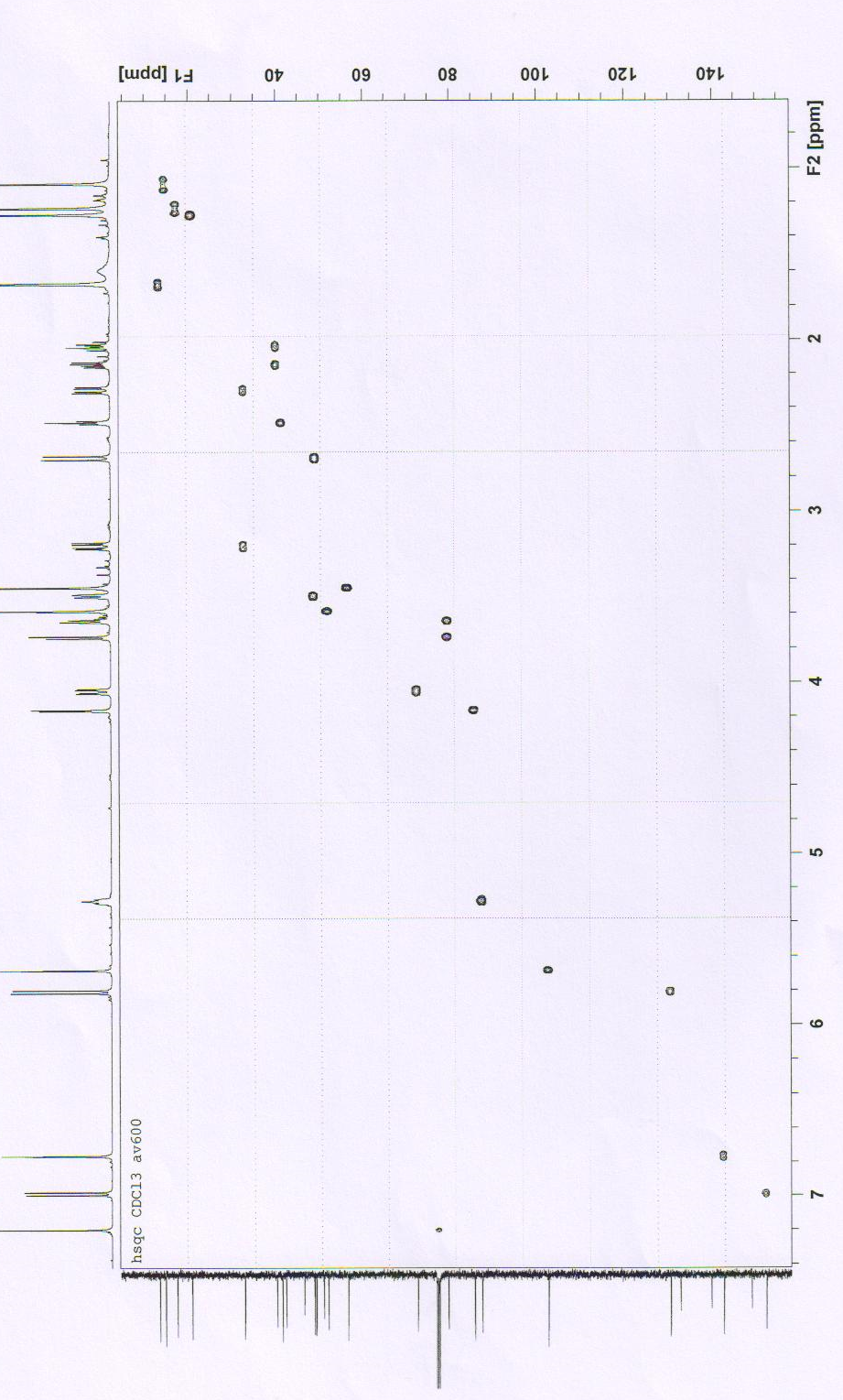


**Figure 4S.** ^1^H-^1^H COSY spectrum of compound **1**.

**
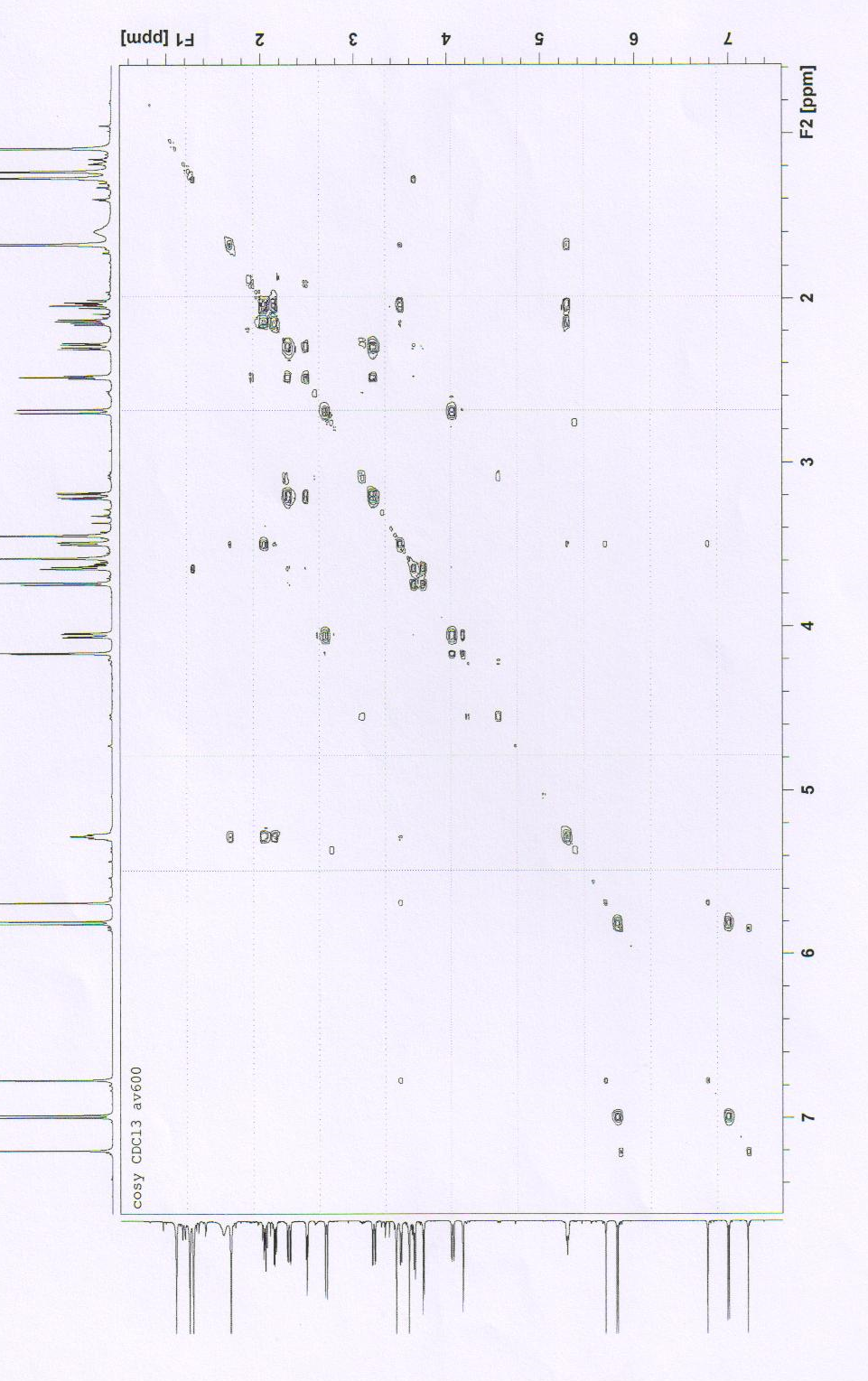
**

**Figure 5S .** HMBC spectrum of compound **1**.

**
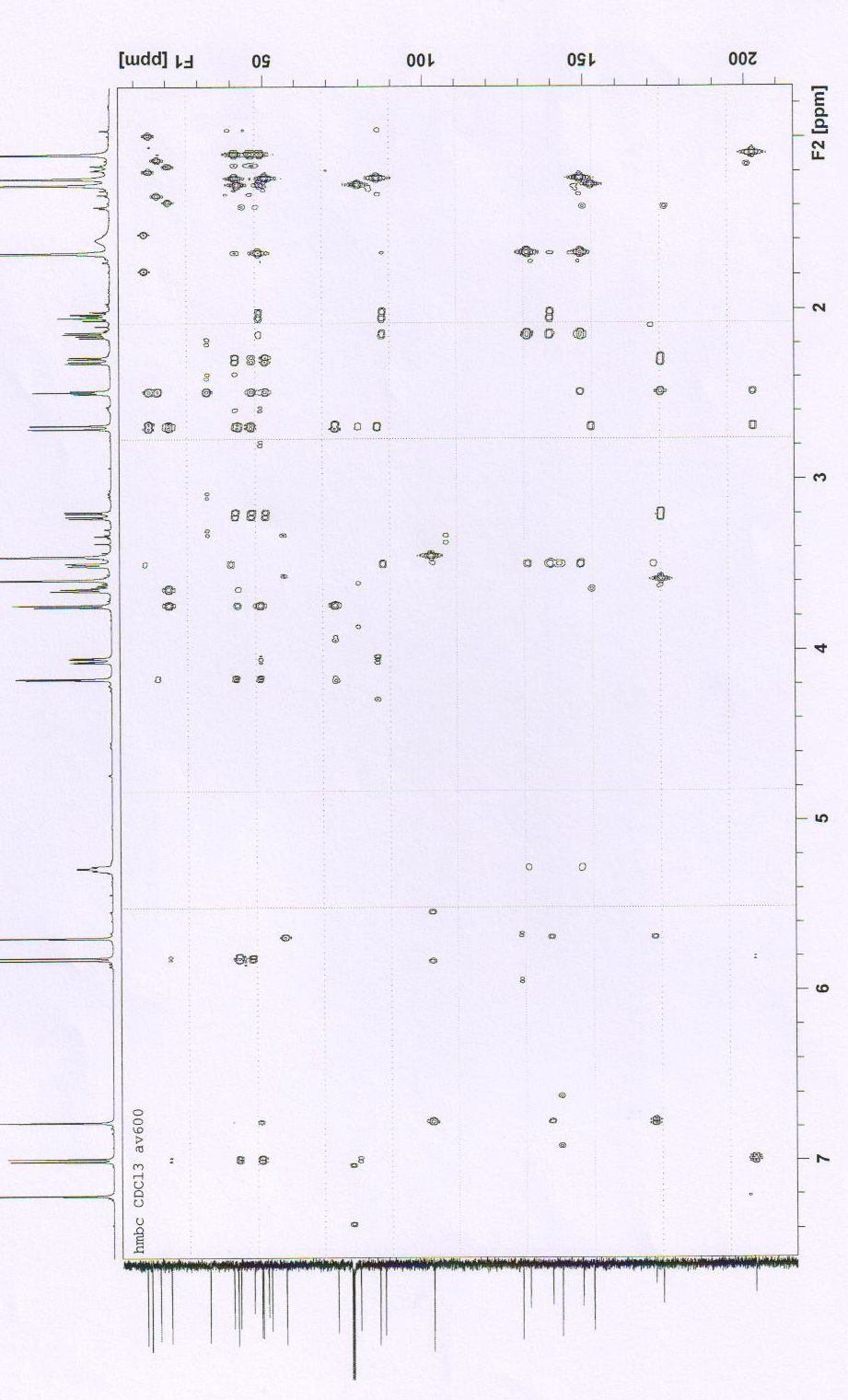
**

**Figure 6S.** ROESY spectrum of compound **1**.

**
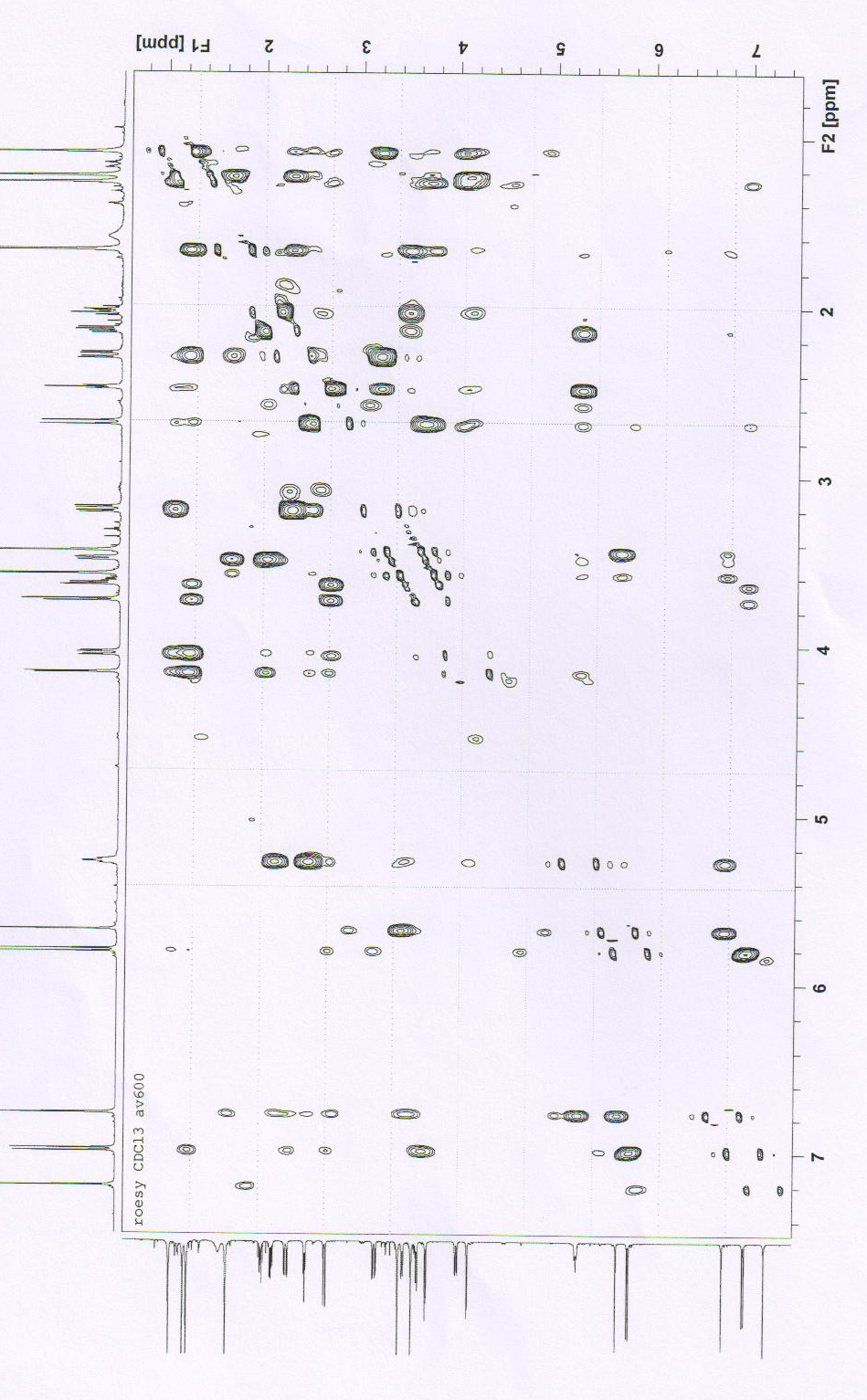
**

**Figure 7S.** HREIMS spectrum of compound **1**.


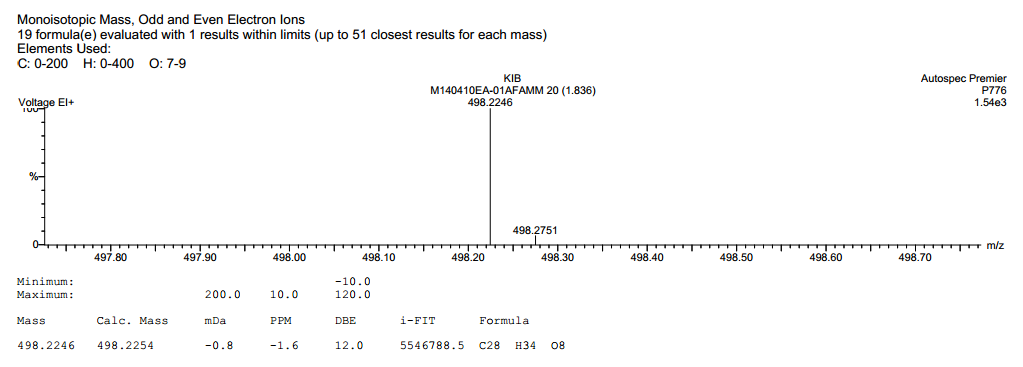


**Figure 8S.** ^1^H NMR spectrum of compound **2**.

**
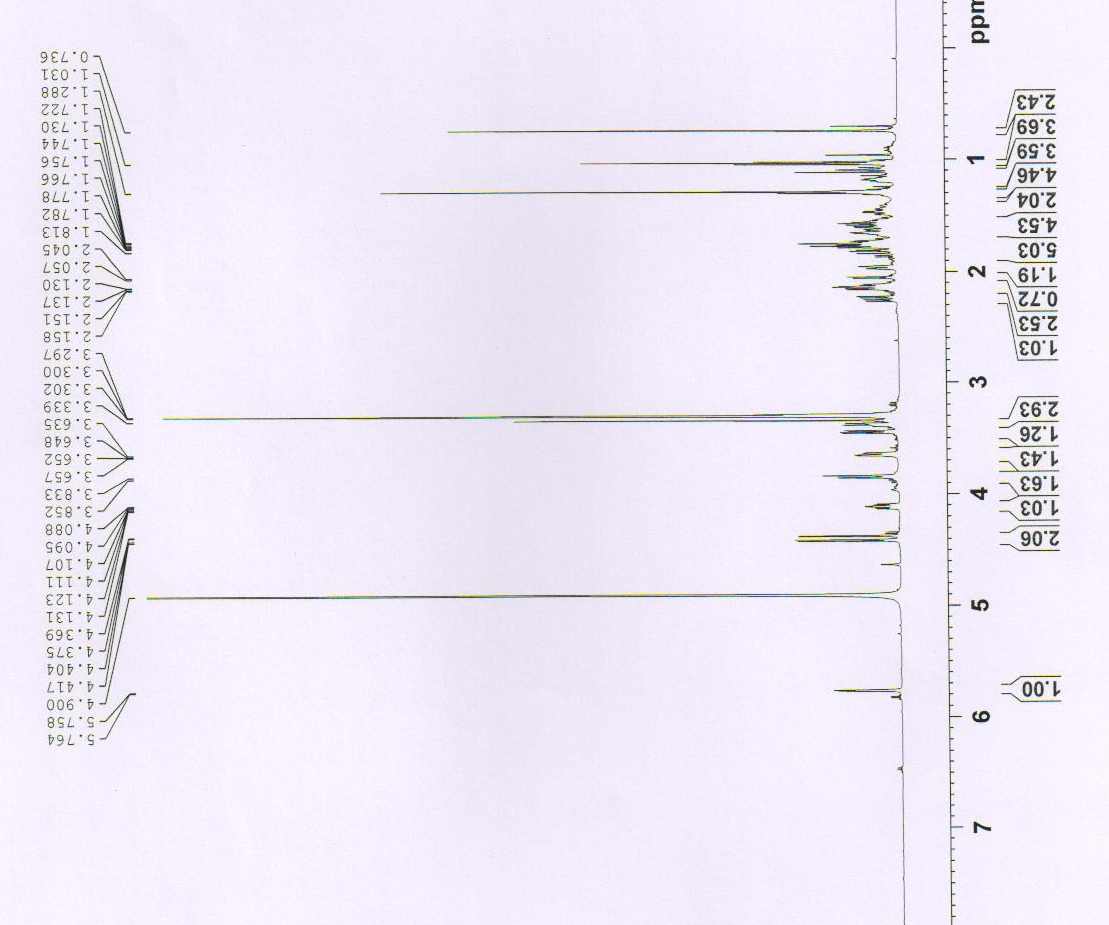
**

**Figure 9S.** ^13^C NMR spectrum of compound **2**.


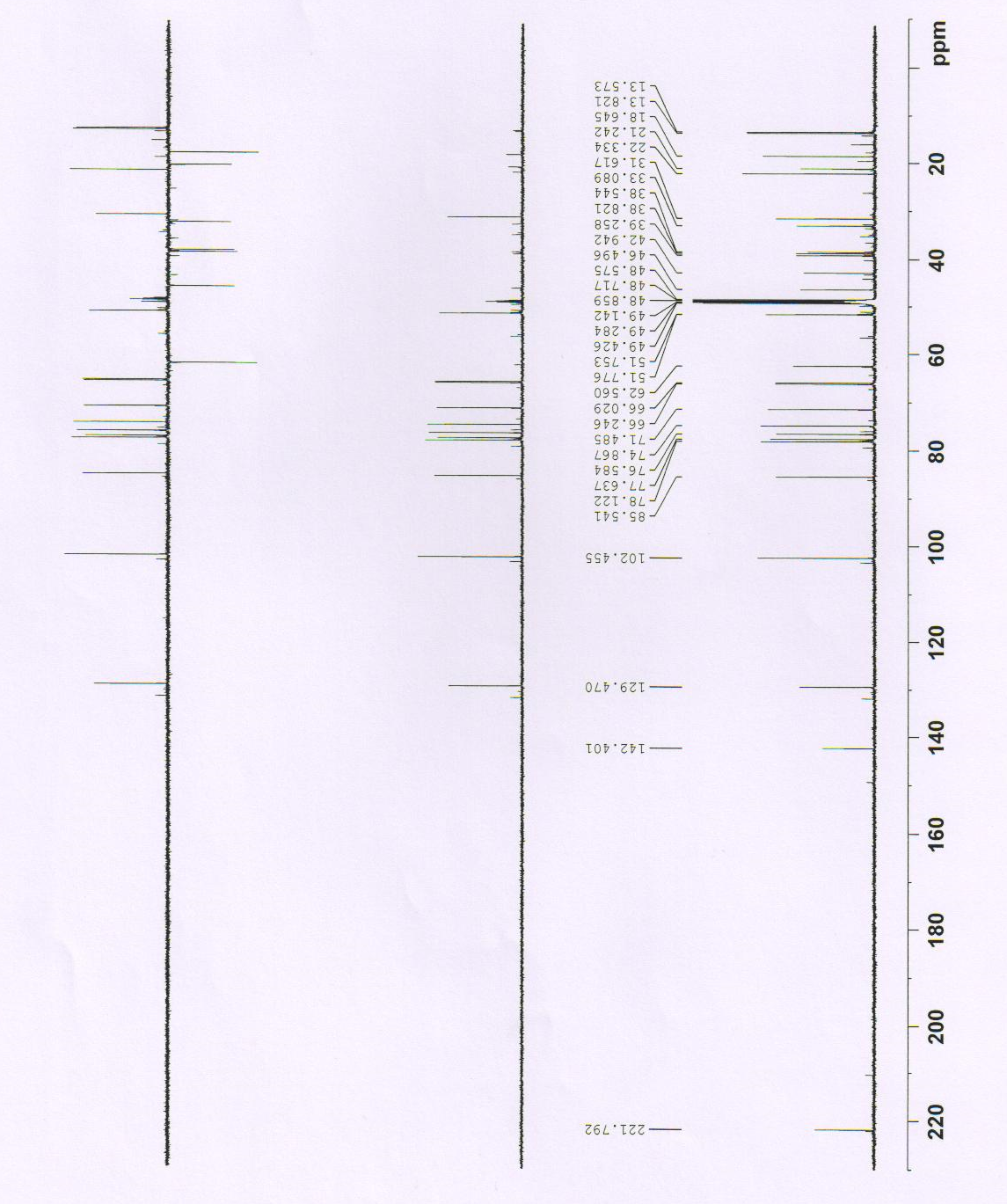


**Figure 10S.** HSQC spectrum of compound **2**.


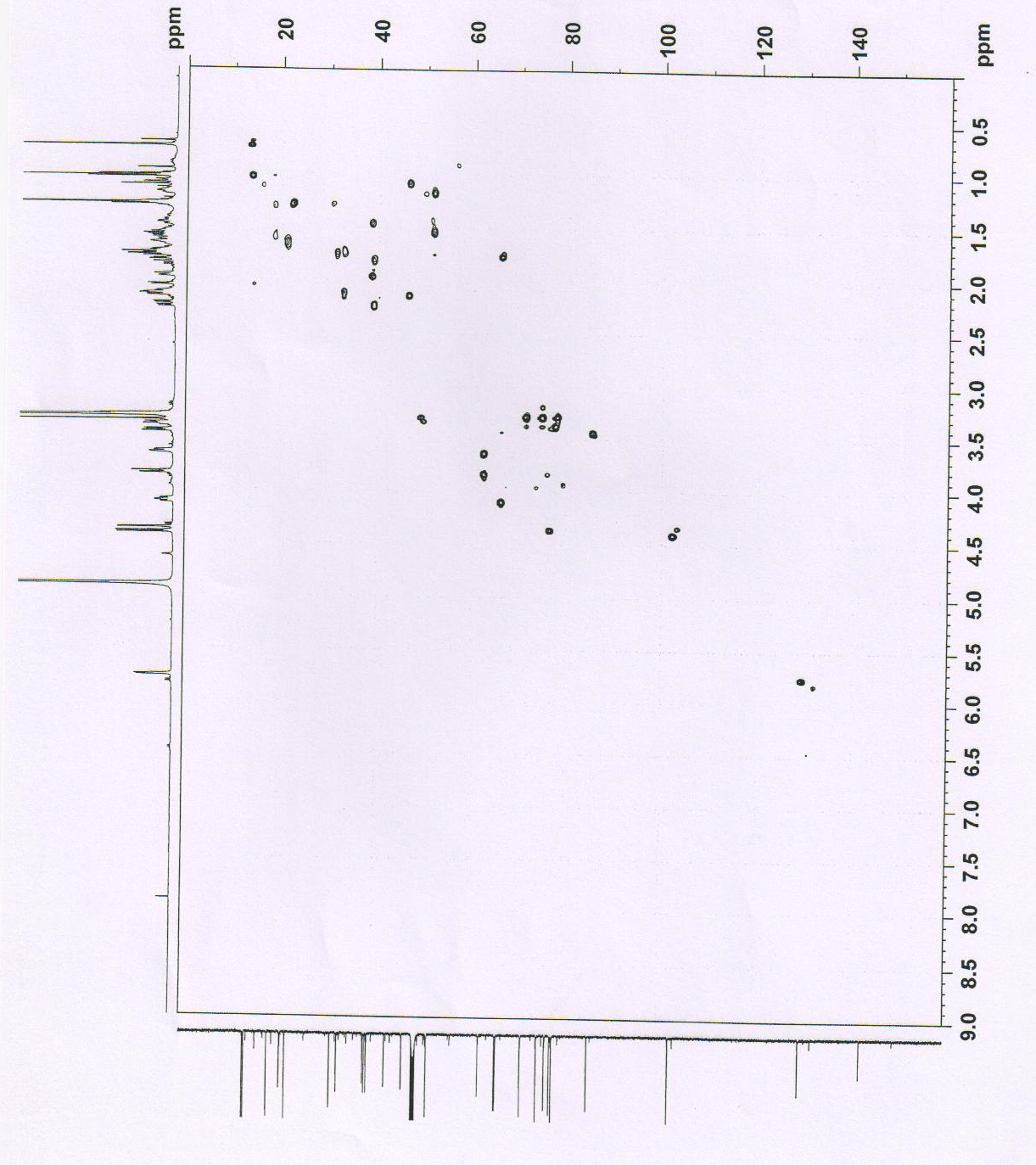


**Figure 11S.** COSY spectrum of compound **2**.


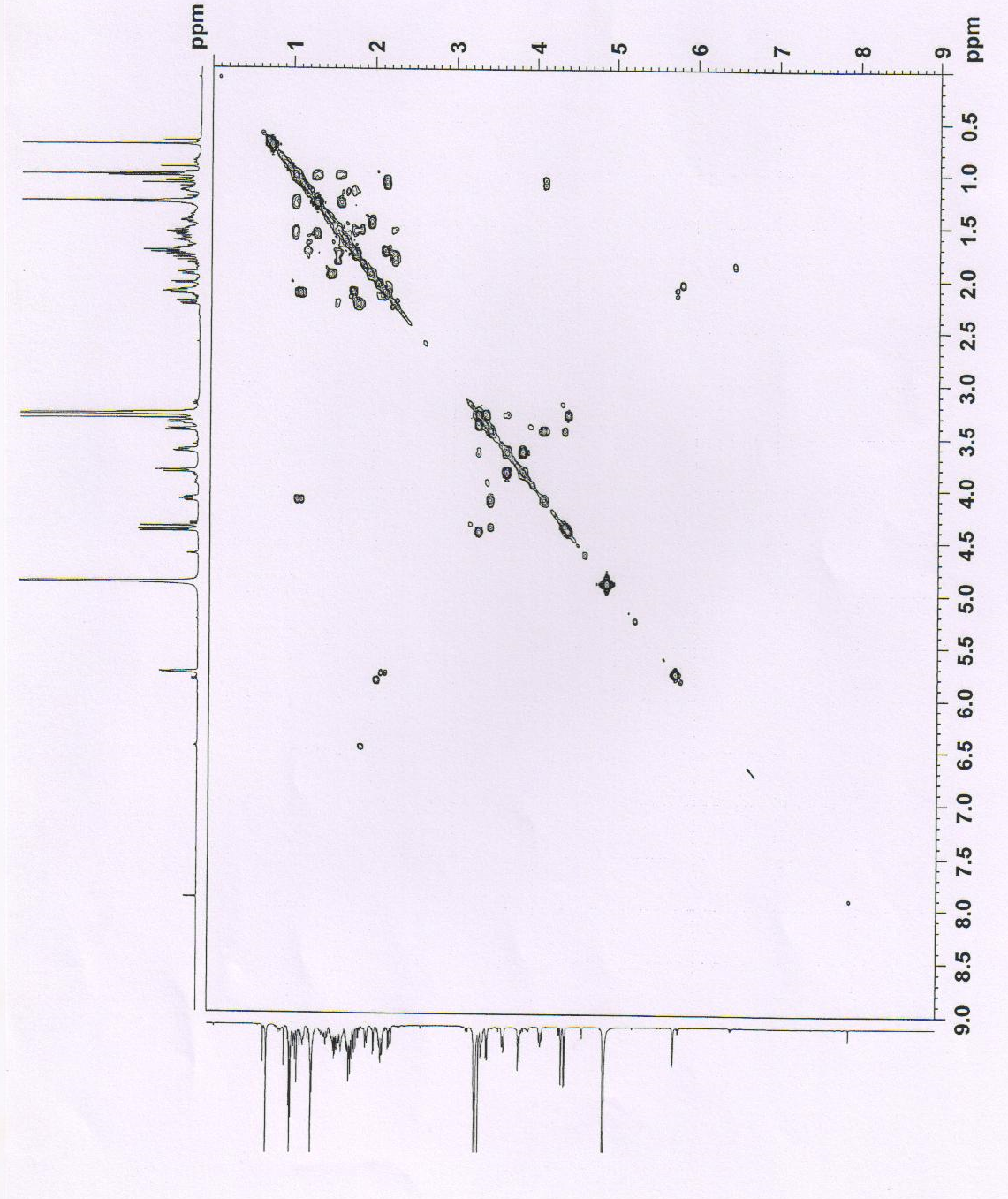


**Figure 12S.** HMBC spectrum of compound **2**.

**
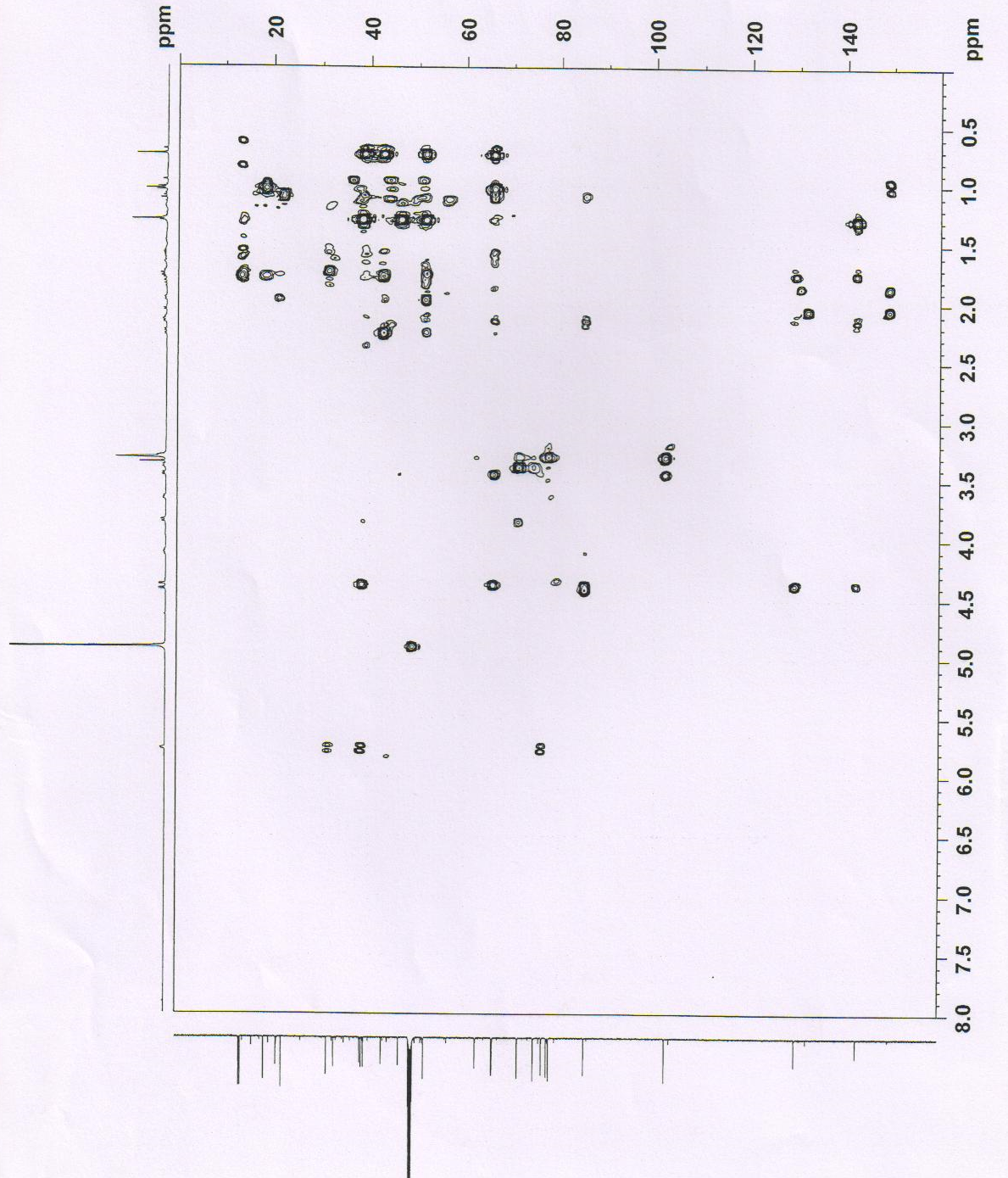
**

**Figure 13S.** ROESY spectrum of compound **2**


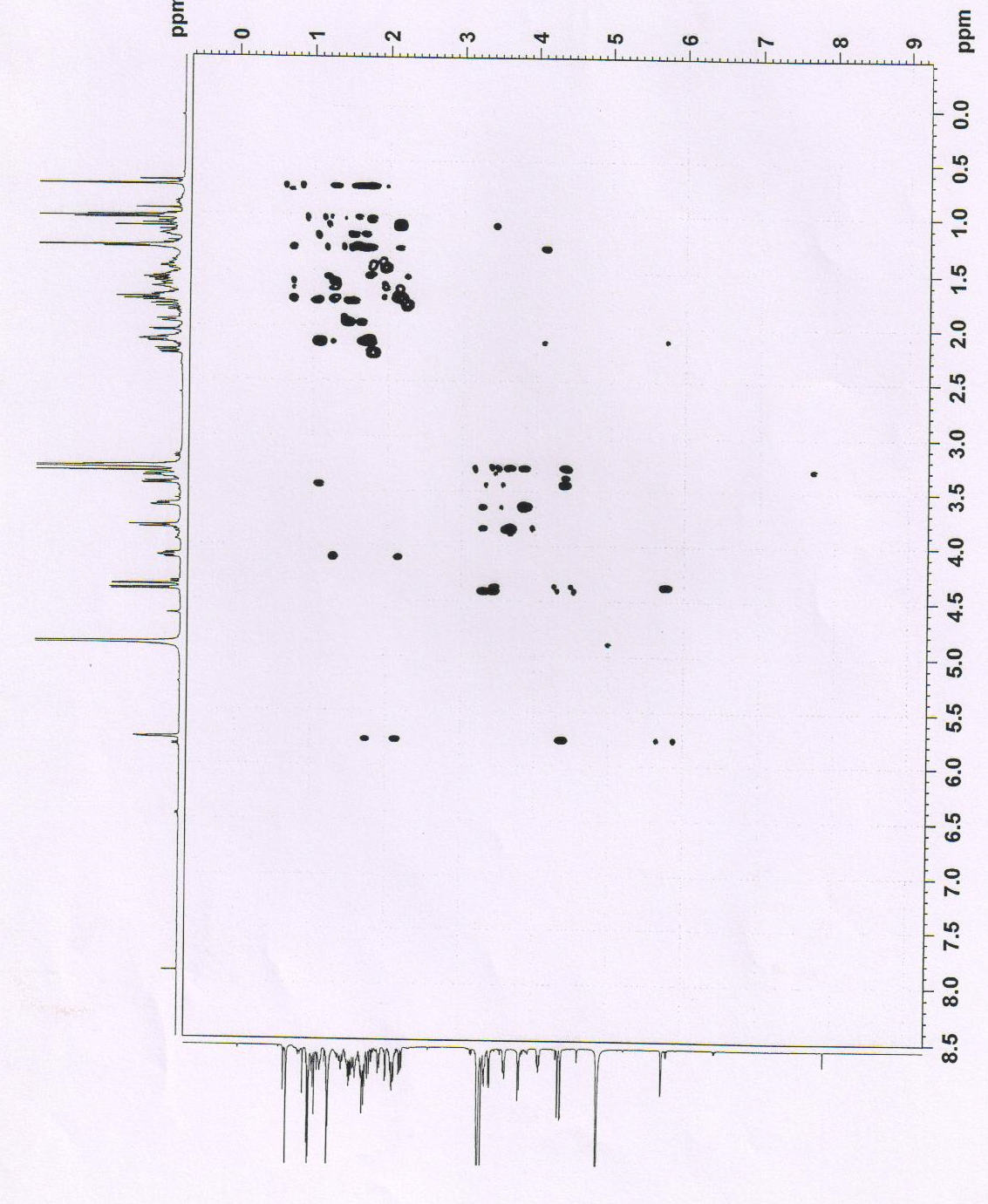


**Figure 14S.** HREIMS spectrum of compound **2**.


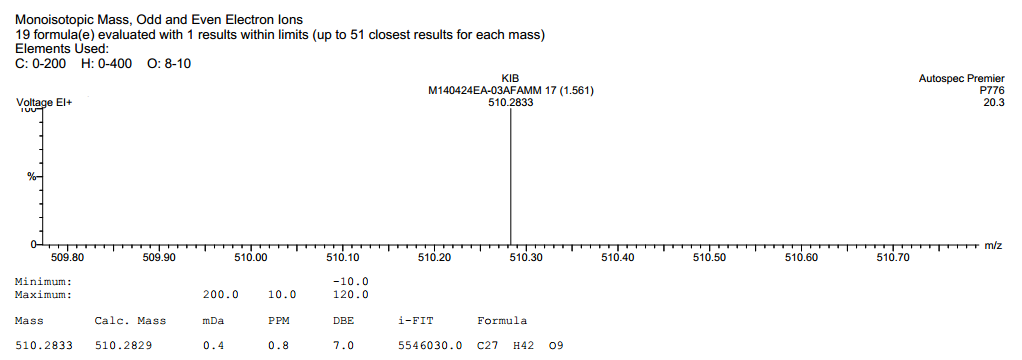

Supplement: Supplementary file 1 — (DOC 16118 kb) [file 13659_2014_42_MOESM1_ESM.doc]
